# Comparing health service use before and after transition to a supported housing model for clients who experience severe and persistent mental illness in southwestern Ontario, Canada

**DOI:** 10.23889/ijpds.v9i5.2528

**Published:** 2024-09-18

**Authors:** Richard G. Booth, Melody Lam, Cheryl Forchuk, Annie Yang, Salimah Z. Shariff

**Affiliations:** 1 Arthur Labatt Family School of Nursing, Western University; 2 ICES Western, London Health Sciences Centre Research Institute; 3 London Health Sciences Centre Research Institute

## Objective and Approach

Beginning in 2018, a provincially-funded custodial housing model in Ontario, Canada transitioned to a supported housing model that allowed more autonomy for clients who experience severe and persistent mental illness (SPMI). The objective of this study was to compare health service use before and after transition to the new Community Homes for Opportunity (CHO) program in southwestern Ontario between 2017 and 2019. Information about clients who transitioned to the CHO program were obtained from the Ontario Ministry of Health and Long-Term Care and linked to health administrative data at ICES. Rates of emergency department (ED) visits, primary care visits, and specialist visits in the one year before and after implementation of the CHO program were compared using conditional Poisson models.

## Results

Of the 368 clients, approximately 40% were female and the mean age was 57 years old. Compared to before the transition, clients had more primary care visits (rate ratio [RR] = 1.21; 95% CI: 1.07-1.37) and specialist visits (RR = 1.33; 95% CI: 1.11-1.60) within the year after transition to the CHO program. There was no significant change in the use of emergency department visits following the transition (RR = 1.20; 95% CI: 0.96-1.50).

## Conclusions

Supported housing may be associated with greater use of primary and specialist care for clients who experience SPMI.

## Implications

The new supported housing model may encourage clients to use more personalized health care, according to their specific needs. Such personalized support is crucial for clients with complex health care needs.

